# Around the Hospitals

**Published:** 1894-07-07

**Authors:** 


### Around the Hospitals.

[Notice to the Managers of Institutions.—Special space is now reserved for the insertion of notices of hospital meetings and festivals. The Editor requests that all notices may reach The Hospital Office, 428, Strand, at least one week before the date of each meeting.]

**The Salford Royal Hospital.**—The Salford Hospital has abolished the system of letters of recommendation since the beginning of the year; an enlightened and disinterested policy which entails extra expense and extra work to the institution and its staff. In spite of a careful inquiry, most of those applying for free relief have been declared incapable of providing for their medical treatment. Another instance of the spirit of progress which dominates the management is the adoption of the uniform system of accounts as required by the London Hospital Sunday Fund of all hospitals participating in its benefits. The whole report of this institution shows a desire to afford information, and is most excellently set forth. We have sometimes to complain of our difficulties when dealing with hospital reports, but it is a far pleasanter task to accord praise where it is due, as in the case of the work of the secretary of the Salford nstitution. Seeing that the public may well entrust funds to good management, we hope to be able in the near future to congratulate the hospital on greater financial prosperity. Some handsome legacies and donations have fallen in, amounting to over £4,000, but the income during 1893 fell short of the requirements by £709. Few new subscribers are recorded, and the recent canvass has not reached the expectations of the board. The patients have numbered 1,229 in the hospital, while 9,764 patients have attended at the out-patients' department, and accidents have numbered 5,836. The cost per patient was 2s. 4½d., and the average cost per bed amounted to £42 14s. 3½d. These sums show a considerable reduction in expenditure, the cost per bed in 1892 reaching £52 15s. 1⅞d.

**The Queen Louise Children's Hospital, Copenhagen.**—At this hospital, in the welfare of which the Queen of Denmark takes such an active interest, there were last year received 395 children (226 boys and 169 girls). After completed treatment (cured or dead) 285 children left the hospital, and 106 children left without their cure being completed (improved or incurable). The number of deaths amounted to 106. The percentage of deaths, arrived at by dividing the number of children who left the hospital into the number of deaths, was 0 265. The average daily number of children was 46·58. For account of the Copenhagen Corporation 312 children were received, and nearly all the children have come from Copenhagen and its immediate neighbourhood. At the "polyclinic" of the hospital 4,013 children have been treated, and the number of consultations at the hospital during the year amounts to 12,367. The branch "polyclinic" in the city has treated 1,179 children. The general state of health during the year has been satisfactory The aggregate income of the hospital has been Kr.89,889, or close upon £5,000.

